# Dural sac cross-sectional area is a highly effective parameter for spinal anesthesia in geriatric patients undergoing transurethral resection of the prostate: a prospective, double blinded, randomized study

**DOI:** 10.1186/s12871-020-01059-x

**Published:** 2020-06-03

**Authors:** Wei Bing Wang, Ai Jiao Sun, Hong Ping Yu, Jing Chun Dong, Huang Xu

**Affiliations:** Department of Anesthesiology, The Affiliated AnQing Hospitals of Anhui Medical University, 352th, Renming Road, AnQing, 246003 AnHui province China

**Keywords:** Transurethral resection of the prostate, Geriatric, Spinal anesthesia, Bupivacaine, Dural sac cross-sectional area

## Abstract

**Background:**

Spinal anesthesia is optimal choice for transurethral resection of the prostate (TURP), but the sensory block should not cross the T10 level. With advancing age, the sensory blockade level increases after spinal injection in some patients with spinal canal stenosis. We optimize the dose of spinal anesthesia according to the decreased ratio of the dural sac cross-sectional area (DSCSA), the purpose of this study is to hypothesis that if DSCSA is an effective parameter to modify the dosage of spinal anesthetics to achieve a T10 blockade in geriatric patients undergoing TURP.

**Methods:**

Sixty geriatric patients schedule for TURP surgery were enrolled in this study. All subjects were randomized divided into two groups, the ultrasound (group U) and the control (group C) groups, patient receive either a dose of 2 ml of 0.5% isobaric bupivacaine in group C, or a modified dose of 0.5% isobaric bupivacaine in group U. We measured the sagittal anteroposterior diameter (D) of the dural sac at the L3–4 level with ultrasound, and calculated the approximate DSCSA (A) according to the following formula: *A* = *π*(*D*/2)^2^, ( *π* = 3.14). The modified dosage of bupivacaine was adjusted according to the decreased ratio of the DSCSA.

**Results:**

The cephalad spread of the sensory blockade level was significantly lower (*P* < 0.001) in group U (T10, range T7–T12) compared with group C (T3, range T2–T9). The dosage of bupivacaine was significantly decreased in group U compared with group C (*P* < 0.001). The regression times of the two segments were delay in group U compared with group C (*P* < 0.001). The maximal decrease in MAP was significantly higher in the group C than in group U after spinal injection (*P* < 0.001), without any modifications HR in either group. Eight patients in group C and two patients in group U required ephedrine (*P* = 0.038).

**Conclusions:**

The DSCSA is a highly effective parameter for spinal anesthesia in geriatric patients undergoing TURP, a modified dose of local anesthetic is a critical factor for controlling the sensory level.

**Trial registration:**

This study was registered in the Chinese Clinical Trial Registry (Registration number: ChiCTR1800015566).on 8, April, 2018.

## Background

Benign prostatic hyperplasia has a high incidence rate about 60% among males aged more than 60 years [1]; whereas the rate is up to 90% among patients around 80 years [2]. The high comorbidity rate also directly affecting perioperative morbidity and mortality after TURP [3, 4]**.**

Because of the pain signal from bladder distension travels along the T11 to L2 sympathetic fibers. The stretch sensation of the bladder is carried by the S2 to S4 parasympathetic fibers. Considering this innervation, the height of the regional blockade level up to T10 is sufficient for TURP operation. A higher level of blockade may mask the pain upon perforation of the prostatic capsule. Intrathecal anesthesia is optimal choice for TURP, but the height should not cross the T10 level. The factors such as concentration and volume are the major factors affecting the distribution of local anesthetics after spinal injection [5]**.**

Hypotension is the major risk after the spinal injection. The systemic vascular resistance may decrease by 25% in elderly patients, whereas it may decrease only by 15–18% in normovolemic healthy patients [6]**.** Because the functional of critical organ and compensate ability for stresses are decreased, it is harmful for geriatric patients to inject more local anesthetics [7]**.** Thus, it is important to optimize the dosage of spinal anesthetics for geriatric patients.

Spinal anesthesia can reduce the stress response relate to surgery [8], and recognize the TUR syndrome early. The patient can complain of shoulder or periumbilical pain with spinal anesthesia level is less than T10 [9]**.** It is necessary to diagnosis and effective management the TUR syndrome timely [10]**.** In a case report, the authors emphasize that it is very important to diagnosis and treatment the TURP syndrome early, the patient have not been found developed hyponatremia until decreased to 90 mmol l^− 1^ under general anesthesia during a TURP procedure [11]**.** The patients can clearly describe the early features of TUR syndrome when patient is conscious, so spinal anesthesia is therefore desirable to facilitate early recognition [10]**.**

The major challenges of spinal anesthesia for geriatric patient are the changes of anatomical and physiological. Some of anatomical irregularities and physiological changes such as reduction in the number of neurons, especially spinal canal stenosis, etc. always associated with increasing age. The blockade level increases after epidural anesthesia and spinal anesthesia [12, 13]**.**

Previous study shown that the depth of intrathecal spaces can accurate prediction by ultrasound imaging [14]**.** A > 30% reduction in the DSCSA and sagittal anteroposterior diameter has been observed in patients with lumbar spinal stenosis [15]**.** The DSCSA is a more sensitive measurement parameter to predict lumbar central canal spinal stenosis [16]**.** Thus, measuring the sagittal anteroposterior diameter of the dural sac with ultrasound can evaluate the degree of lumbar central canal spinal stenosis.

Optimal blockade levels by intrathecal anesthesia is favorable for TURP operation for adequate blockade of the stimulation of bladder traction and less hypotension and bradycardia by too high thoracic block. For geriatric patients, sensory blockade up to T10 is favorable for adequate anesthesia with less hypotension and bradycardia. Most anesthesiologists may reduce the dosage of intrathecal anesthetics to prevent too high blockade by experience. However, as lumbar central canal spinal stenosis is more frequently found in geriatric patients, we hypothesized that local anesthetics would spread more cephalad with a limited space. With goal to achieve T10 sensory blockade in patients receiving TURP operation, we modified the dose of bupivacaine according to the decreased ratio of the DSCSA. By comparing with controlled groups receiving 10 mg of 0.5% isobaric bupivacaine, we analysis the levels of sensory blockade, and the changes of mean arterial blood pressure (MAP) and heart rate (HR).

The purpose of present study is to determine the hypothesis that if DSCSA is an effective parameter to modify the dosage of spinal anesthetics to achieve a T10 blockade in geriatric patients undergoing TURP.

## Methods

### Design

We conducted a prospective, double blinded, randomized study to measure the sagittal anteroposterior diameter of the dural sac by ultrasound for geriatric patients aged more than70 years undergoing TURP with spinal anesthesia, and then calculated the DSCSA, optimizing the dosage of local anesthetic according to the decreased ratio of the DSCSA.

### Subjects and setting

Sixty geriatric patients schedule for TURP surgery were enrolled in this study. The medical ethical committees of The Affiliated AnQing Hospital of Anhui Medical University approved this study on 26, December, 2017, and the study was registered in the Chinese Clinical Trial Registry (Registration number: ChiCTR1800015566). The informed consent were written by all patients.

The exclusion criteria of this study as following: local infection at the puncture site, administrated with anticoagulants, intracranial hypertension, and patients who did not to accept spinal anesthesia. Relative contraindications included some neurologic diseases (e.g. multiple sclerosis), lower limbs pain, and so on.

### Study protocol

All subjects were randomized divided into two groups, the ultrasound (group U, *n* = 30) and the control (group C, *n* = 30) groups, according to the random number table generator by computer (prepared by AJS).

All patients transported to the operating room, where they were subjected to standard monitoring electrocardiography (ECG), and pulse oximetry (SPO_2_). The MAP and HR were monitored throughout the operation also.

All intrachecal anesthesia operation was performed by the same anesthesiologist (an associate chief physician of anesthesiology). Epidural puncture was located at the L 3–4 intervertebral space, the spinal needle was inserted into the subarachnoid space after successfully epidural puncture, 2 ml of 0.5% isobaric bupivacaine was injected in group C, and group U received a modified dose according to the DSCSA measured by ultrasound when cerebrospinal fluid (CSF) appeared in the needle hub. Then, the spinal needle was withdrawn.

### Measurements

MAP and HR were measured every 2.5 min during surgery in the first 30 min after spinal injection and, then every 15 min until the end of the study.

The cephalad sensory level was measured via a cold alcohol cotton swab every 5 min until 30 min after the spinal injection and, then every 15 min until regression below L4. Ten minutes after the spinal injection, if the sensory blockade level was below T10, remifentanil 0.1–0.2 μg kg^− 1^ min^− 1^ was treated intravenous continuous infusion to maintain a sufficient analgesia level.

The motor block level was measured by modified Bromage scale every 5 min until 30 min after the spinal injection and, then every 15 min until complete motor recovery occurred. Modified Bromage scale: 0: able to move the hip, knee, ankle, and toes; 1: able to move the knee, ankle, and toes; 2: able to move the ankle and toes; 3: only able to move the toes; and 4: unable to move the hip, knee, ankle, and toes.

The local anesthetics was prepared by an anesthesia assistant (HPY), and she did not assessed all patients. Another anesthesiologist (JCD or HX) assessed the cephalad sensory level and measured the Bromage scale, who remained blinded to the local anesthetics.

If the systolic blood pressure decrease more than 30% compare with the baseline, intravenous 5 to 10 mg ephedrine was treated, and a HR of less than 45 beats min^− 1^, intravenous 0.5 mg atropine was treated.

We assessed and recorded the variables, the maximal sensory level, sensory level regression by 2 dermatomes, and complete motor block recovery.

### Image analysis

**Fig. 1:**
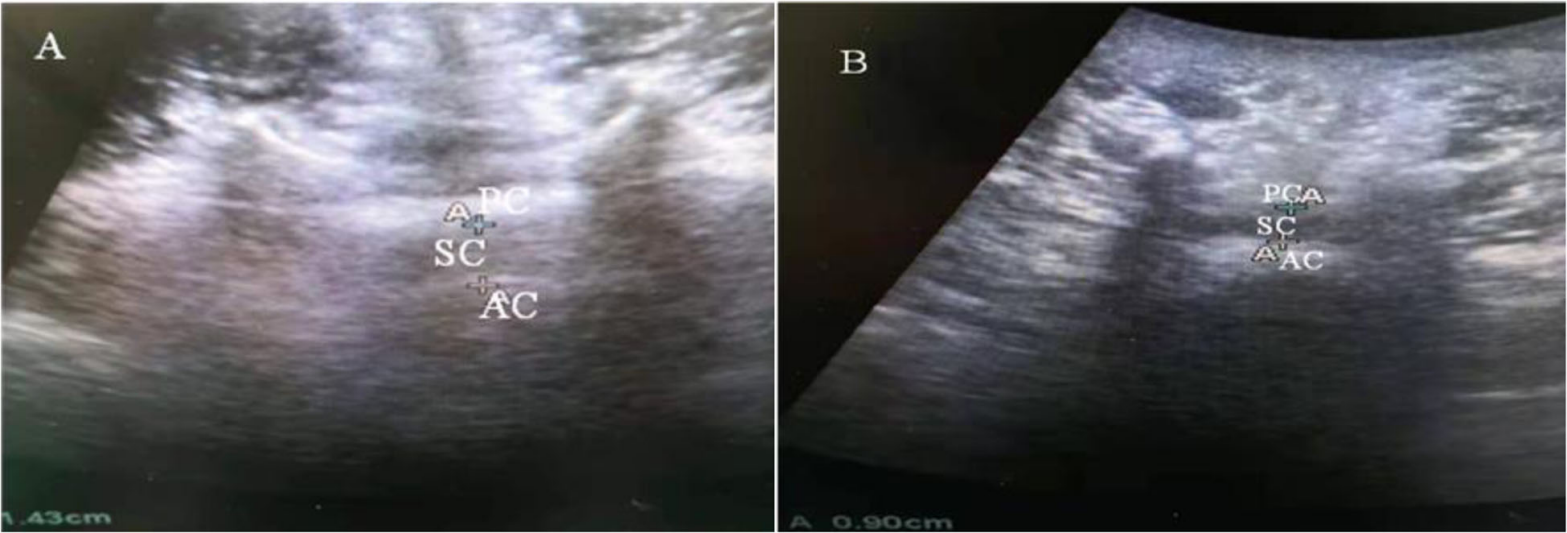
SC = spinal canal, AC = anterior complexus, including the posterior longitudinal ligament and vertebral ligament, PC = posterior complexus, including the ligamentum flavum and endorhachis

A previous study indicated that 10 mg of 0.5% intrathecal bupivacaine provided a sufficient level of sensory blockade for elderly patients undergoing TURP [17]. Lim YS et al. [16] showed that the average DSCSA was 151.67 ± 53.59 mm^2^ in the control group (without lumbar central canal spinal stenosis) and 80.04 ± 35.36 mm^2^ in the lumbar central canal spinal stenosis group. Thus, we hypothesized that the dosage would be more excessive for some geriatric patients with lumbar central canal spinal stenosis, and that would be a greater cephalad spread of local anesthetics. We measured the sagittal anteroposterior diameter (D) of the dural sac at L3–4 with ultrasound, and calculated the approximate DSCSA (A) according to the formula: *A* = *π*(*D*/2)^2^, ( *π* = 3.14). For example, to determine the DSCSA (Fig. 1), the diameter (the distance between AC and PC) of the dural sac was measured. The diameter shown in the picture A was 14.3 mm, and the DSCSA was 160.5 mm^2^. However, another diameter shown in the picture B was 9.0 mm, and the DSCSA was 63.6 mm^2^.

### Modified dose of bupivacaine

We confirmed that the primary DSCSA was 150 mm^2^ and that the primary dose of bupivacaine was 10 mg. The modified dose of bupivacaine was adjusted according to the decreased ratio of the DSCSA compared with the primary DSCSA of 150 mm^2^. For example, if we measured the D of the dural sac to be 10 mm, then, A = 78.5 mm^2^, and the decreased in the ratio of DSCSA was 48% ((150–78.5)/150 = 0.48), thus, the modified dose of bupivacaine was decreased by 48%, so 5.2(10–10*0.48 = 5.2) mg bupivacaine was spinally injected.

### Statistical analysis

We using G*Power software to estimate the sample size. Taking into consideration the results of previous studies, we set an alpha as 0.05 and a power as 0.8, the result of software shown that at least 26 patients in each group, therefore, 30 patients in each group was a sufficient sample size.

The various parameters were statistically analysed using the SPSS 17.0 (SPSS Inc., Chicago, IL, USA). Continuous data were evaluated with independent samples t-test, sensory level with Mann-Whitney U test, and frequency data with Chi square test. *P* < 0.05 was considered statistically significant.

## Results

**Fig. 2:**
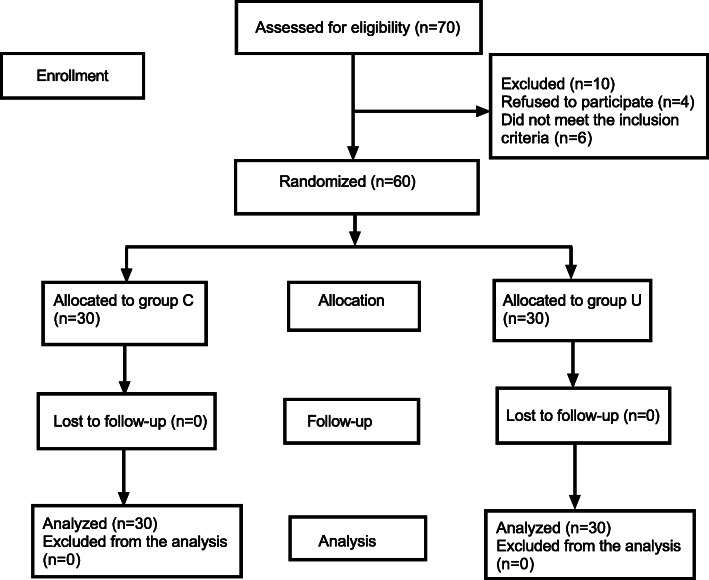
CONSORT flow diagram

The patients flow diagram of this study is shown in Fig. 2. Seventy patients were assessed for eligibility for this study, four patients refused to participate this study and six patients did not meet the inclusion criteria, and finally 60 patients were randomly divided into two groups, 30 patients in each group.

Demographic characteristics (age, weight, height), ASA classification, duration of surgery, dosage of bupivacaine and DSCSA were compared in two groups (Table 1). The dosage of bupivacaine was significantly decreased (*P* < 0.001) in group U compared with group C.

**Table 1:** Demographic characteristics, ASA status, duration of surgery, dose of anesthetics and DSCSA (Mean ± SD)

	Group C (*n* = 30)	Group U (*n* = 30)	*P*-value
Age (years)	78.3 ± 5.8	77.4 ± 5.5	0.555
Height (cm)	169.0 ± 6.9	169.6 ± 7.2	0.771
Weight (kg)	65.9 ± 8.9	66.0 ± 8.6	0.977
ASA(I / II / III)	16/10/ 4	18 /9/ 3	0.855
Duration of surgery (min)	92.4 ± 17.8	86.2 ± 19.3	0.354
DSCSA( mm^2^)	106.8 ± 8.2	102.5 ± 7.6	0.924
Dose of bupivacaine (mg)	10.0 ± 0.0	6.7 ± 1.6	< 0.001

*ASA* American Society of Anesthesiologists, *DSCSA* dural sac cross-sectional area

**Fig. 3:**
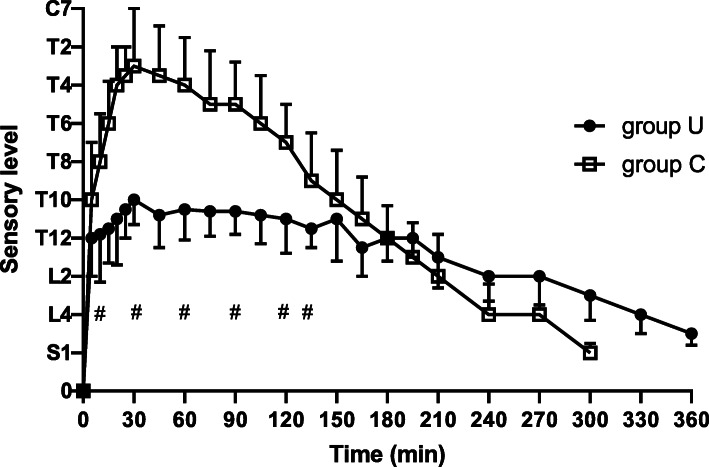
Evolution of the sensory level over time in two groups, the sensory level were higher from 10 to 135 min after spinal injection in group C than in group U, #*P* < 0.001

The main data of the spinal anesthesia were collected and shown in Table 2. The evolution of the sensory blockade level were shown in Fig. 3. The cephalad spread of the sensory blockade level was significantly lower (*P* < 0.001) in group U (T10, range T7–T12) compared with group C (T3, range T2–T9). The regression times of the two segments were delay in group U than in group C (*P* < 0.001, Table 2).

**Table 2:** Main data of the spinal block

	Group C (*n* = 30)	Group U (*n* = 30)	*P*-value
Maximal sensory level	T3(T2-T9)	T10(T7-T12)	< 0.001
Onset time to maximal sensory block (min)	25.2 ± 10.4	26.3 ± 12.2	0.636
Regression by 2 segments (min)	102.0 ± 28.2	156.1 ± 42.3	< 0.001
Total motor recovery (min)	186.2 ± 58.0	175.1 ± 44.2	0.620

**Fig. 4:**
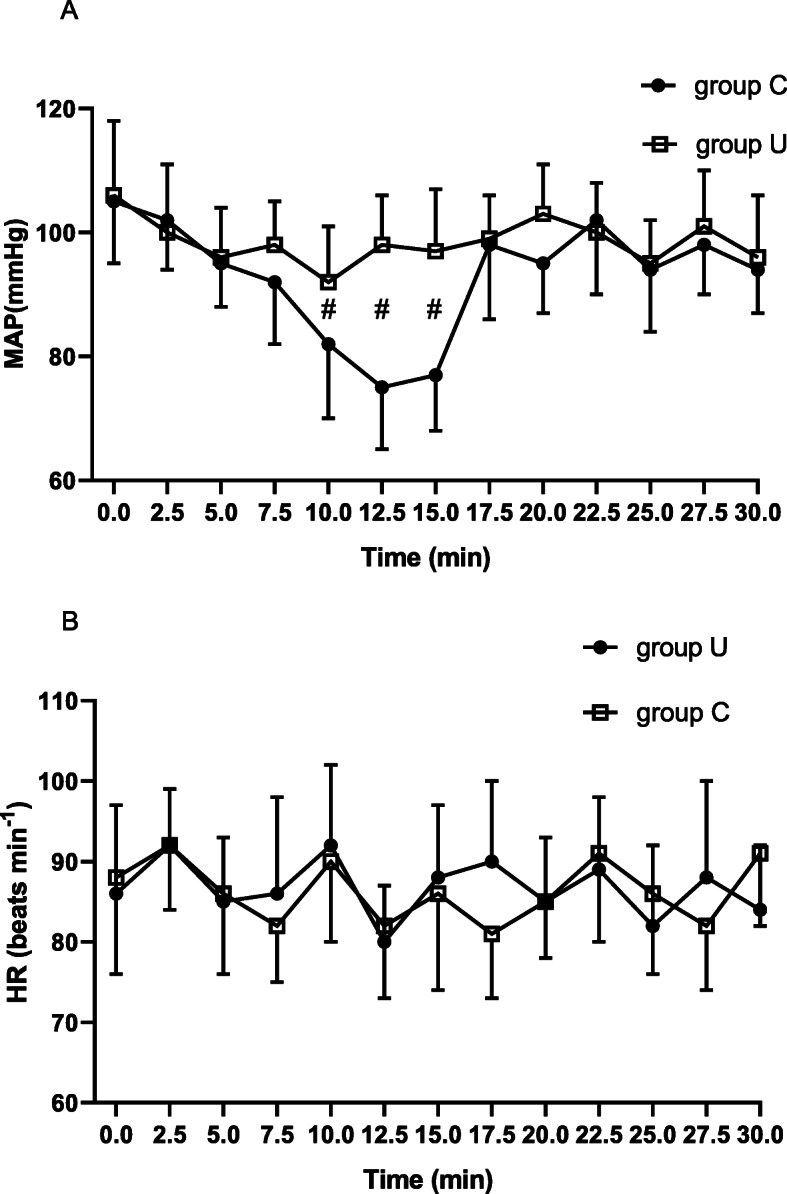
**a** The changes of the MAP over the first 30 min after spinal injection in two groups, the MAP were significent decreased at the time of 10 min, 12.5 min and 15 min in group C than in group U, ^#^*P*< 0.001. **b** The changes of the HR over the first 30 min after spinal injection in two groups, there were no significent different at each time in two groups

Figure 4a and b represents the evolution of the MAP and HR in the first 30 min of the study, respectively. The maximal decrease in MAP was significantly higher in the group C than in group U after spinal injection (*P* < 0.001, Table 3). Eight patients in group C and two patients in group U required ephedrine (*P* = 0.038, Table 3).

**Table 3:** Hemodynamic characteristics

	Group C (*n* = 30)	Group U (*n* = 30)	*P*-value
Baseline MAP (mmHg)	105.3 ± 10.2	106.0 ± 12.0	0.865
Baseline HR (beats min^−1^)	82.3 ± 10.2	86.0 ± 9.2	0.726
Maximal decrease in MAP (% of baseline value)	26.2 ± 13.3	12.2 ± 10.1	< 0.001
Number of patients receiving ephedrine	8	2	0.038

*MAP* mean arterial pressure, *HR* heart rate

## Discussion

The purpose of this study was to determinate the relationship between the DSCSA and the dose of local anesthetic. Two groups with the same demographic date were compared but injected with different doses of bupivacaine to show the highest spreads level up to T3 in group C and T10 in group U, (Table 2.). The results confirm our hypothesis, a higher cephalad spread would occur without a modified dose in group C, and a higher cephalad spread would not occur with a modified dose according to the DSCSA in group U.

A questionnaire based on Japanese population for predicting lumbar stenosis, the results shown that the incidence increased with age, with an incidence of 1.7–2.2% between ages 40 and 49, and of 10.3–11.2% between ages 70 and 79 [18]**.** In our study, the dosage of bupivacaine was significantly lower in group U than in group C (*P* < 0.001, Table 1.). This finding may be related to the lumbar stenosis in some geriatric patients. Low dose of local anesthetic is the important reason to limit the higher cephalad spread.

Degenerative spondylosis is a significant etiology of lumbar spinal stenosis. Wear-and-tear changes and trauma, among other factors, such as lumbar disc herniation, ligamentum flavum hypertrophy, osteoporosis, posterior longitudinal ligament ossification, the spinal venous plexus proliferation, and congenital stenosis, which in turn will cause spinal stenosis, occur with aging [19]**.** Therefore, the traditional dose of local anesthetics may be excessively for patients with lumbar spinal stenosis. It is unclear whether this is the case for patient with lumbar spinal stenosis, so it is important for anesthesiologists to control the sensory level for each patient. The greater the cephalad spread is, the higher the incidence of hypotension and bradycardia.

The MAP was significantly decreased in group C compared with group U (Table 3.). Previous studies [20] shown the same results as our finding, and it may be related to widely sympathetic block caused by excessive bupivacaine with the higher cephalic sensory level in group C, so it needs more ephedrine to maintain the MAP in group C than in group U (*P* = 0.038, Table 3).

The regression times of the two segments were significantly longer in group U than in group C (*P* < 0.001, Table 2). The spread and eliminate of local anesthetics after spinal injection could be explained by its pharmacokinetics. The arachnoidal and dural was determinate the eliminate of local anesthetics, and it’s concentration gradient was determinate by vascular absorption in epidural venous plexus. Simultaneously, the subarachnoid space venous plexus also absorbed local anesthetics. If the blockade level is high accordingly the dosage of bupivacaine to block a segment is low. It require a greater meningeal surface to eliminate local anesthetics if the block level is spread greater.

Patients undergoing TURP are generally older and have various comorbidities [4]. It is important to restrict the blockade level to maintain the hemodynamic instability after spinal injection. Although there are many factors that determines the sensory level, including the dosage of local anesthetic and not by the block position, anesthetic volume, or concentration [21–23]. Therefore, the dosage of spinal injection should be decreased in order to restrict the blockade level. Most anesthesiologists think that decreased the dosage of spinal injection may induce insufficient spinal block. There were many studies to balance between the low dose of spinal injection and insufficient spinal block, the coadministration of additives such as opioids or dexmedetomidine was together with spinal injection to improve the block quality [17, 24]. However, the complications such as bradycardia, hypotension, vomiting, nausea, pruritus and excessive sedation were emerged after the coadministration [25–27]. Compare with the normal population, the DSCSA was a 30% decrease in patients aged more than 70(Table 1), so we suggest a 30% reduction of bupivacaine for patients aged more than 70, especially measure DSCSA for each patient around 80 before performing spinal anesthesia. The benefits of DSCSA-adjusted dosage for intrathecal anesthesia includes less hypotensive episodes and less ephedrine to treat them.

In addition, a higher cephalad blockade level is not required for TURP surgery, and a T10 is sufficient sensory level. The sympathetic such as pelvic plexus and hypogastric plexus, and parasympathetic such as S_3_ and S_4_ dominate prostate and bladder. Because of the urethral internal sphincter and external sphincter are dominated by the pelvic plexus and the pudendal nerve respectively, both of the nerves were block, and then the urethral sphincter would be adequately relaxed and the endoscope could pass smoothly. A previous study shown that T12–L1 sensory block was sufficient for TURP to avoid discomfort due to irrigation-induced bladder distension, but there were more many patients required analgesics during the postoperative period [3]**.** In our study, only one patient who showed abdominal discomfort with sensory level regression to <T10 because the duration of surgery exceeded 2 h in group U, remifentanil 0.1μg kg^− 1^ min^− 1^ was treated intravenous continuous infusion for abdominal discomfort during the operation, 20 min after, the surgery was finished.

The most anatomical change in geriatric patients is lumbar central canal spinal stenosis. The most frequently applied criteria are the measurement of the anteroposterior diameter of the cross-sectional area of the dural sac and of the osseous spinal canal for lumbar central canal spinal stenosis [28]**.** Thus, the analysis of the DSCSA is very important for anesthesiologists to evaluate the degree of lumbar central canal spinal stenosis in each patient. Currently, the optimal cut-off value of 111.09 mm^2^ for the DSCSA has a high sensitivity (80.0%) and specificity (80.8%) for predicting lumbar central canal spinal stenosis [14]**.** This optimal cut-off value is less than that of some patients without lumbar central canal spinal stenosis. Therefore, greater cephalad spread results from an excessive dose without regulation according to the DSCSA in group C.

There were several limitations of the current study. Unlike magnetic resonance imaging (MRI), ultrasound cannot be used to accurately discriminate the AC from PC, thus, some errors may arise in the sagittal anteroposterior diameter of the dural sac. Second, the DSCSA is not a normal circle, so, the DSCSA we calculated according to the formula is only an approximate value. Third, the research population included a small number of lumbar central canal spinal stenosis patients. The demographic characteristics, such as weight, height and degree of obesity still various.

Despite these limitations, the results are important for spinal anesthesia in geriatric patients to compare the DSCSA and the dose of local anesthetics.

## Conclusions

The DSCSA is a highly effective parameter for spinal anesthesia in geriatric patients undergoing TURP, a modified dose of local anesthetic is a critical factor for controlling the sensory level.

## Data Availability

The datasets are available from the corresponding author on reasonable request.

## Abbreviations

TURP: Transurethral resection of the prostate
DSCSA: Dural sac cross-sectional area
MAP: Mean arterial blood pressure
HR: Heart rate
CSF: Cerebro-spinal fluid
NIBP: Noninvasive blood pressure
ECG: Electrocardiography
SpO_2_: Peripheral capillary oxygen saturation
MRI: Magnetic resonance imaging
